# Self-Testing of Vision in Age-Related Macula Degeneration: A Longitudinal Pilot Study Using a Smartphone-Based Rarebit Test

**DOI:** 10.1155/2015/285463

**Published:** 2015-06-01

**Authors:** Christina Winther, Lars Frisén

**Affiliations:** Department of Clinical Neuroscience and Rehabilitation, Institute of Neuroscience and Physiology, The Sahlgrenska Academy, University of Gothenburg, 413 45 Gothenburg, Sweden

## Abstract

*Purpose*. There is a need for efficient self-tests of vision in patients with neovascular age-related macula degeneration. A new tablet/smartphone application aiming to meet this need is described and its performance is assessed in a longitudinal pilot study. *Materials and Methods*. The new MultiBit Test (MBT) employs segmented digits defined by rarebits, that is, receptive field-size bright dots briefly presented against a dark background. The number of rarebits per digit segment was varied in a cyclic fashion, in preset steps. There were no fixation demands. Twenty-eight patients with neovascular AMD of varying severity were monitored for an average of 30 weeks. Test scores were evaluated on an individual basis, by contrasting observed trends with the clinical status recorded at independently scheduled clinical examinations. *Results*. Serial plots of MBT results revealed gradual improvement after successful antineovascular treatment. Recurrences were signalled by gradual deteriorations of results. Test results remained stable during clinically stable time intervals. MBT results agreed well with clinical assessments whereas an acuity test performed at chance level. The MBT was well accepted by all subjects. *Conclusions*. The MBT appears to have a good potential for effective self-testing of vision in AMD and merits large-scale studies. Exploration of MBT performance with other forms of macula conditions may be worthwhile.

## 1. Introduction

Management of patients with macula edema is a major logistic challenge. First, patients show considerable variations in their responses to treatment and in rates of recurrence. Second, key examination resources like optical coherence tomography and retinal angiography are finite. Third, treatment is expensive. Management might best be optimized by involving active monitoring by the patients themselves, by arranging for suitably spaced self-tests of vision. Ideally, such tests not only should alert caregivers to deterioration of results but should also provide measures of rates and magnitudes of change to aid priority assignments.

There is a long tradition of recommending self-testing by means of plain Amsler metamorphopsia grids. Unfortunately, Amsler test results are subjective and notoriously difficult to interpret [1]. Acuity tests are easier to evaluate but require both rigorous control over test conditions and outside assistance to check responses for accuracy. These limitations apply equally to clinical charts and to the multitude of look-alikes that are available on Internet websites and as software applications or “apps” for tablets and mobile phones [2]. Further, acuity tests exhibit considerable intersession variability and some patients report pronounced short-term variations of vision [3–7]. Acuity tests appear to have a limited sensitivity to macula edema [8].

There are several alternative ways to monitor vision in macula conditions [9]. Those that rely on dedicated hardware, for example, microperimeters, preferential hyperacuity perimeters, and adaptometers, may be too expensive to find widespread use among patients. Electronic tablets and so-called smartphones, on the other hand, are readily available to many patients and could serve as useful platforms for novel, dedicated self-tests for macula edema [10, 11]. For maximum utility, such tests should be fairly tolerant to environmental variables like test distance, they should be doable without outside assistance, and they should be capable of transmitting results for remote monitoring. Additional desirable properties include small or no demands on fixation stability and the use of ever-new test target combinations, to prevent false gains from memorization.

A recently described shape discrimination test, the myVisionTrack, appears to meet the above demands [10, 11]. Using a forced-choice test paradigm, results are automatically scored without outside assistance. Set up for advanced remote monitoring, the test has been shown to be capable of discriminating groups of subjects with different stages of age-related macula degeneration (AMD). Its capacity to identify changes over time in individual cases is currently not known.

The present study explored the utility of another type of test, namely, a test that employs receptive field-size dots or “rarebits.” Rarebit testing was devised with the specific aim of uncovering low degrees of neurovisual damage, where conventional tests often fail [12]. The rarebit name refers to the test target's information content, which is minuscule compared with the information overload contained in conventional test targets. Normal eyes are expected to see essentially all rarebit probes, in central and peripheral vision alike. Conversely, eyes that have suffered losses of receptive fields or damage to their upstream connections will miss some probes, in relation to the severity of damage. Several studies of various aspects of rarebit testing have reported good sensitivity and specificity for a variety of retinal and visual pathway lesions, good reproducibility, and good acceptance by tested subjects [8, 12–18].

**Figure 1:**
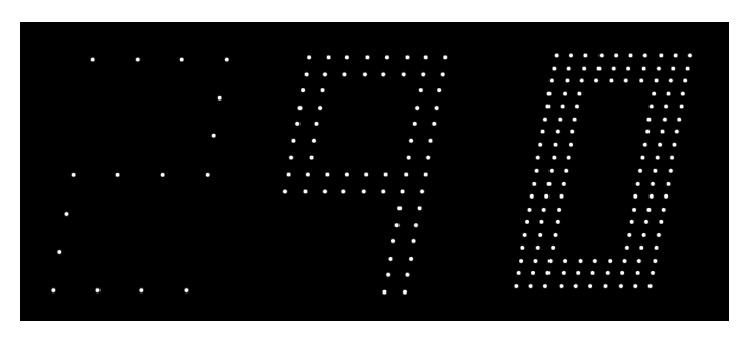
Examples of segmented digits defined by different numbers of rarebits per segment (from left to right, 4, 16, and 30). For a dynamic demonstration, see 〈http://www.oft.gu.se/webdiagnos/multibit/multibit.html〉.

The original rarebit tests are impractical for self-testing. A new presentation format, namely, segmented digits, may have a better potential.  Figure 1 shows examples of segmented digits. These types of targets have the unique property of predictable normal limits. A normal eye needs no more than three rarebits to recognize a segment, even if briefly flashed, whereas eyes that have lost receptive fields need larger numbers. There are no fixation demands. Initially developed in a personal computer setting, the multiple rarebit test principle has been shown to perform significantly better than conventional clinical tests in a variety of conditions, including macula edema [8, 13].

Here, we describe a new multiple rarebit test version in the form of a smartphone/tablet application named the MultiBit Test (MBT). The MBT allows remote monitoring of results from nonassisted self-tests. We present longitudinal observations from a small series of patients being monitored for AMD with choroidal neovascularization (CNV). Test performance was evaluated on an individual basis, by contrasting trends among observed MBT results with objective features documented during independently scheduled clinical examinations. The aims of this first pilot study were to obtain a general impression of test performance and to secure the information needed to plan a long-term, large-scale multicenter study. The main outcome measures were concordance of trends and aspects of sensitivity and specificity.

## 2. Materials and Methods

Patients were recruited from the AMD/CNV treatment programme of the Retina Unit at the Sahlgrenska University Hospital, a tertiary-care center. Exclusion criteria were the bilateral presence of currently inactive lesions judged to run a small risk of recurrence, the presence of additional, non-maculopathic causes of visual loss, and inability to participate in conventional acuity testing. Twenty-eight patients partook in the study. Those who had the same type of lesions in both eyes (active or inactive) provided results from the least involved eye only whereas those who had different types of lesions provided results from both eyes. A total of 36 eyes qualified for study.

All clinical examinations were performed by author Christina Winther. Examination intervals were decided on an individual basis, depending on the response to any previous antineovascular treatment and on current disease activity as judged from biomicroscopy, ocular coherence tomography (OCT), and retinal angiography. Lesions showing edema and/or leakage were judged active, otherwise inactive. Currently active lesions were usually treated within a few days of examination, using ranibizumab in accordance with the manufacturer's recommendations (Novartis, Basel).

Twenty control subjects were recruited primarily from patients' relatives or other accompanying persons. All controls were examined once only, in the same way as the patients, excluding angiography. To avoid intereye dependencies, results from one eye only were used for statistical analysis. The selection of right-eye results was arbitrary.

All subjects were naïve to rarebit testing. All gave informed consent. The examinations adhered to the tenets of the Declaration of Helsinki. Ethics Committee approval was obtained.

### 2.1. The MultiBit Test

The MBT was developed by author Lars Frisén for iPhone and iPod Touch tablets with high-resolution Retina screens (Apple, Cupertino, CA). The latter type of device was used here because of its lower price and lower operating costs. At a laptop viewing distance (0.5 m), each screen pixel subtended 0.5′ or about 80% of that of a foveal cone. The full screen subtended 10.0 × 5.7°. Screen brightness was forced (in software) to 80% of maximum. Surround illumination was outside investigator control as actual tests were performed in home settings but patients were instructed to seek total darkness.

Segmented digits were generated in the manner previously described for the DigitStep test, the personal computer precursor to the app [8]. The test digits subtended 40 × 50′, equivalent to 0.1 decimal (20/200 Snellen) optotypes. They were built up by single-pixel rarebits in a segmented format as exemplified in Figure 1. Numbers of rarebits per segment (RPS) were preset at 3, 4, 5, 6, and 7 in test level 1 and 8, 10, 16, 30, and 60 in test level 2. In the interest of saving on test time and maximizing tested areas, digits were always presented in pairs, with identical RPS numbers. Pair members were selected at random and presented in a left-to-right sequence in randomly selected screen locations. The sequential presentations served to prevent crowding effects. The digits were presented for 150 ms each, with a 150 ms blank interval in between, that is, briefly enough to frustrate refixation. Digit pairs were separated by a time interval of 4 seconds, providing ample time for response.

A complete MBT test comprised 3 presentations of the 5 RPS settings contained in the user-selected test level. The test task was to call out all digits seen. Guessing was encouraged. The verbal responses were automatically recorded in an internal audio file. Once all 15 digit pairs had been shown, the app entered scoring mode. Here, the same sequence of 15 digit pairs was displayed in an easily read format and the audio file was played back. Scoring was performed by comparing the digits actually shown with the recorded responses, pair by pair, and tapping appropriately labelled buttons. The percentage of correct responses was calculated automatically at the end of the test and displayed as the test score. Test duration was about 2 min, not counting the time needed for scoring results. Inbuilt instructions guided users throughout the procedure.

MBT devices were provided on loan to the study participants. Following careful instructions, identification of the appropriate test level, and supervised training, participants were asked to perform monocular self-tests under constant conditions at home, in total darkness, at least twice a week, using their reading glasses, with occlusion of the nontested eye. Those who had access to a wireless local area network and were willing to take on emailing of test results were instructed accordingly. Otherwise, participants were asked to bring their test devices to their scheduled clinical appointments, where downloading of stored results took place.

### 2.2. Visual Acuity

Visual acuity was assessed at all clinic visits using a transilluminated Early Treatment Diabetic Retinopathy Study (ETDRS) acuity chart (Precision Vision, La Salle, IL), using full ametropia correction. Each correctly read test letter provided a score of 1, for a maximum of 100.

### 2.3. Statistical Analyses

Test scores were evaluated on an individual basis and were contrasted with outcomes of clinical examinations performed at independently scheduled intervals. The main outcome measures, concordance between clinical ratings and vision test outcomes, were assessed by means of linearly weighted kappa statistics. Sensitivity and specificity were analysed using receiver-operating characteristic (ROC) curves. The analytical tool (MedCalc Software v 12.7.7.0, Ostend, Belgium) also provided estimated specificity at fixed sensitivity levels as well as estimated sensitivity at fixed specificity levels, with bootstrapped (1,000 iterations) confidence intervals.

## 3. Results

**Figure 2:**
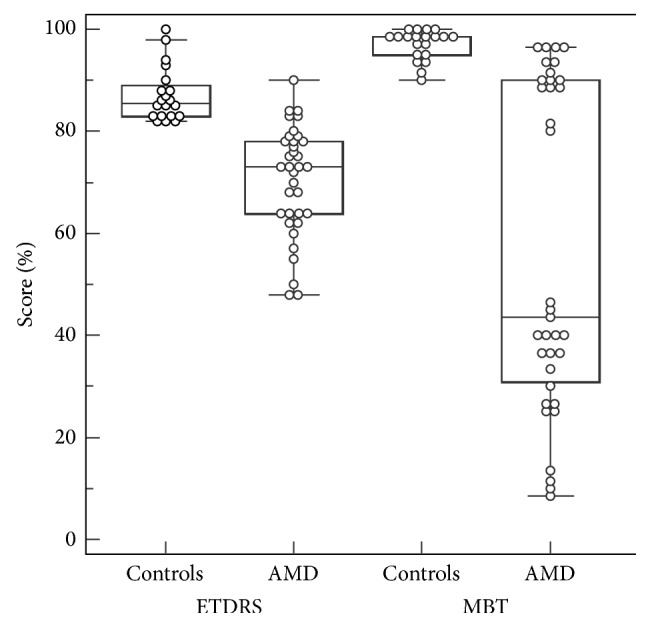
Box-and-whisker plots of ETDRS and MBT results at study entry.

At the time of entry into the study, the 28 patients had a mean age of 76 ± 7 (SD) years. The 20 normal control subjects had a mean age of 68 ± 7 years. All subjects were Caucasian; 55% were females. Among the eligible study eyes, 26 had some degree of macula edema whereas 10 had no edema.  Figure 2 presents overviews of ETDRS and MBT results at entry, revealing a wide range of visual deficits.

The patients returned for renewed clinical evaluations at mean intervals of 63 days. At each follow-up, results were compared with those of the nearest preceding visit. In all, 144 intervisit intervals or epochs were available for analysis. Twenty-six epochs were clinically judged to exhibit impairment, 50 epochs were judged to exhibit improvement, and 68 epochs were judged stable. Decisions as to active treatment were based solely on the results of the clinical examinations. Treatment was provided on 72 occasions.

**Figure 3:**
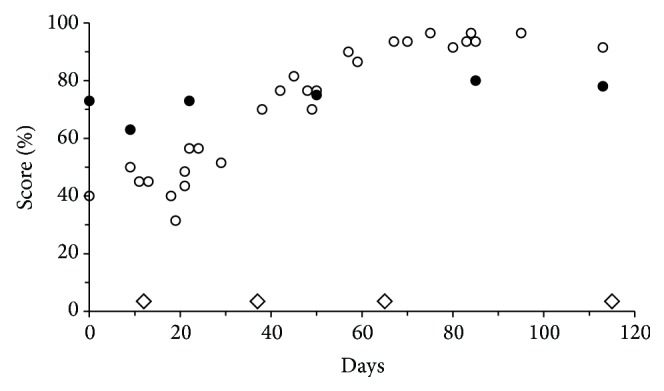
Sequential MBT (*⚪*) and ETDRS (•) results from a patient with newly diagnosed wet AMD in the right eye. The intervals between clinical ETDRS examinations define epochs within which trends of MBT results were assessed. Diamonds (⋄) identify times of treatment.

**Figure 4:**
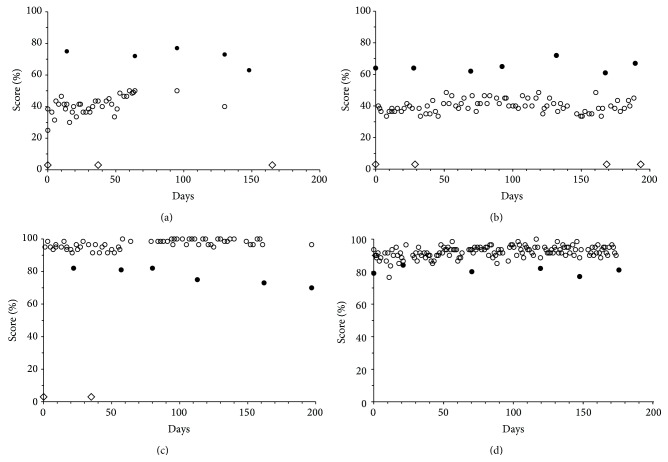
Sequential MBT (*⚪*) and ETDRS (•) results from 3 patients with wet AMD and 1 with dry AMD (d). Diamonds (⋄) identify times of treatment.

The patients made a total of 1203 MBT self-tests during periods averaging 39 weeks in length. Results were plotted against time for each study eye separately.  Figure 3 shows an example of quite pronounced changes. Being closely monitored and treated as needed, most patients showed less dramatic changes, as exemplified in Figure 4. In the figure, filled circles identify both ETDRS results and times of clinical examinations. With MBT, impairments could often be discerned in advance of the preplanned clinical follow-up dates. Following treatment of active disease (in the plots symbolized by open diamonds), MBT scores could be seen to improve, and variation could be seen to decrease, but scores remained consistently subnormal. ETDRS scores generally showed little change.

For formal analysis, all MBT plots were carefully evaluated by subjective inspection, epoch by epoch. Epochs showing trends of decreasing scores, or increasing variation, or both, were rated worse. Epochs showing the opposite evolution were rated better. All other epochs were rated stable. ETDRS results were rated similarly, using direct numerical comparisons of scores. The outcomes of the clinical examinations, which included biomicroscopy and scrutiny of OCT parameters and maps, were summarized in the same manner.

**Figure 5:**
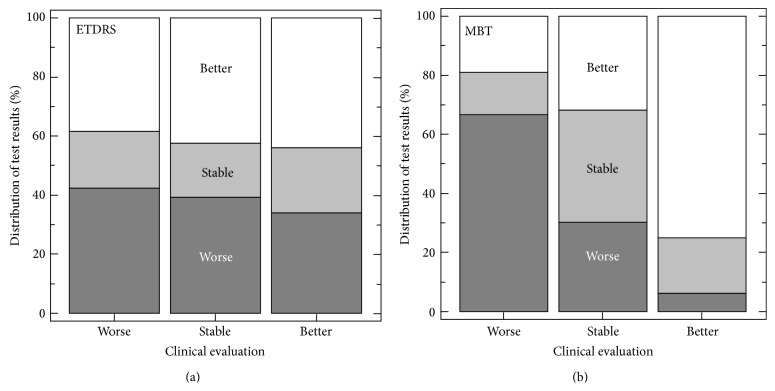
Distributions of clinical versus vision test ratings for 144 follow-up epochs. (a) ETDRS, (b) MBT.

Figure 5 illustrates the concordance of clinical and ETDRS results in (a) and clinical and MBT results in (b). A numerical assessment of agreement was obtained by calculating kappa statistics. By definition, kappa equals 1 in case of perfect agreement whereas kappa 0 indicates chance agreement. For ETDRS, kappa equaled 0.03 (95% confidence interval (CI) −0.09–0.15) and for MBT 0.41 (CI 0.29–0.53). Notably, the ETDRS confidence interval includes 0 whereas the MBT interval neither includes 0 nor overlaps the ETDRS interval.

MBT sensitivity and specificity were illuminated by ROC analysis of entry-point data, when all subjects were naïve to the test task. The area under the curve equaled 0.95 (CI 0.90–0.99). At the 95% specificity level, sensitivity was estimated to be 80% (CI 60–91), at the criterion level < 90. Conversely, at the 95% sensitivity level, specificity was estimated to be 70% (CI 45–90), at the criterion level < 96.

## 4. Discussion

Like its personal computer based predecessor [8], the MBT “app” exhibited good sensitivity and specificity in cross-sectional analysis of the entry-point data. The concordance is gratifying in view of the impossibility to control external factors in real-life self-testing situations. Uncontrolled factors include ametropia correction, viewing distance, background illumination, and various types of environmental distractors. In spite of the uncontrolled external-factor conditions of the present study, the MBT appeared to be highly capable of illuminating visual performance in AMD.

The present finding of limited utility of the ETDRS test was not unexpected as acuity tests have long been known to be quite tolerant to various forms of visual system injury [19]. Plausible explanations relate to an overload of information in both space and time [8, 12] and memorization effects. Furthermore, acuity tests target function in a restricted retinal area only, in and close by the fixation point (or the preferred locus of fixation). Such a narrow focus may be misleading in conditions like AMD, where the disease process is neither confined to the point of fixation nor uniformly distributed throughout the involved area. The MBT bypasses these limitations by minimizing information and employing an extensive test area (10.0 × 5.7°). Furthermore, by employing random positioning of targets within the test area, the MBT does not demand a stable fixation.

Turning to longitudinal aspects of self-testing in AMD, previous information is very limited. The so-called HOME study demonstrated a significantly better outcome for patients who were monitored with preferential hyperacuity perimetry at home compared to patients under routine clinical care. The test's performance on the individual level was not reported, however [20].

The present report demonstrates the possibility to capture gradual changes of scores and score variability with changes in disease activity in individual cases (Figures 3 and 4). Plain eye-balling of serial results often allowed recognition of deterioration in advance of independent physician estimates of optimum clinical examination intervals. Plain eye-balling also allowed confident rejection of occasional out-of-line scores.

One limitation of the present longitudinal analyses concerns their purely subjective nature. This limitation is attributable to the combination of a lack of a theoretical model and a paucity of appropriate analytical tools. Empirical modelling is currently in progress but must be tested against a new data set before its utility can be assessed. Collection of new and larger data sets is one of the goals of a large-scale, multicenter study that is currently being planned. Meanwhile, it is worthy of note that the authors made independent visual evaluations of the result plots. Their evaluations agreed in 141 out of the 144 epochs and only 3 were in need of arbitration.

Another important aspect of agreement concerns that of test results vis-à-vis clinical ratings. For the ETDRS test, agreement was close to chance level, with a kappa statistic not meaningfully different from 0 (see also Figure 5). For the MBT, the kappa statistic did differ from 0 but it did not approach 1, that is, perfect agreement. There is little consensus as to interpretation of intermediate kappa values except that they indicate less-than-perfect agreement. As to the root cause of imperfect agreement, both MBT results and clinical ratings need to be considered. The clinical ratings depended heavily on OCT results and therefore may appear to be highly reliable. However, ocular coherence tomography has its own limitations [21]. Furthermore, the clinical ratings actually constitute subjective summaries of a host of subjective impressions and objective measurements of various morphological features. These features may vary from one part of the macula to another. An assignment of a “stable” clinical grade may be particularly challenging in the presence of active disease. Against this background, it is of little surprise that exclusion of the clinically stable subgroup caused the MBT kappa to rise from 0.41 (CI 0.29–0.53) to 0.57 (CI 0.39–0.75). ETDRS kappa remained at chance level, however (0.06).

The present study cannot inform on optimum self-test intervals. The study participants were asked to perform at least two tests per week but actual frequencies varied not only between patients but also over time (Figures 3 and 4). Nevertheless, a recommendation of twice-weekly testing may strike a constructive compromise between useful diagnostic detail and reasonable demands on patients. Several participants decided on their own to make more frequent observations. Some even made their own spreadsheet analyses.

There were no reports of any difficulties with the MBT test itself. Some subjects encountered occasional problems with electronic transmission of results within wireless local area networks. Retransmission at a later date usually solved this problem. Otherwise, stored results could be accessed at the next clinical appointment. While acceptable for this first pilot study, large-scale usage requires a more robust and more elaborate arrangement. A sophisticated framework can provide additional functions, including reminding patients about missed examinations and actively alerting caregivers to deteriorating test results [10, 11].

The MBT has two inherent limitations, both of which apply to the extremes of the measuring range. At the high RPS end, there is a limit to the number of rarebits that can be contained within the space allotted to the test digits. Therefore, the test may be difficult to apply in cases with advanced visual loss, with optotype acuities worse than approximately 0.2 decimal (20/100 Snellen, 50 ETDRS, 0.7 logMAR). Although it is possible to allot more space to the test digits, there is a trade-off effect in that the perception of dot-defined symbols of large angular subtense may be dominated by dots rather than symbol Gestalts. Another type of limitation applies to the low RPS end, where minute changes of vision may hide in between the two lowest scale steps (3 and 4 RPS). However, this latter limitation can be circumvented by averaging results from repeated measurements. Incidentally, the possibility to select test level appeared superfluous and will be discarded.

The MBT appears to meet the need for a powerful tool for self-testing of neovascular age-related macula degeneration. The test merits closer study in larger-scale trials and also with other types of macula conditions.
